# Neutrophil Extracellular Trap Targeting Protects Against Ischemic Damage After Fibrin-Rich Thrombotic Stroke Despite Non-Reperfusion

**DOI:** 10.3389/fimmu.2022.790002

**Published:** 2022-02-16

**Authors:** Carolina Peña-Martínez, Violeta Durán-Laforet, Alicia García-Culebras, María Isabel Cuartero, María Ángeles Moro, Ignacio Lizasoain

**Affiliations:** ^1^ Unidad de Investigación Neurovascular, Dpto. Farmacología y Toxicología, Facultad de Medicina, and Instituto Universitario de Investigación en Neuroquímica (IUIN), Universidad Complutense de Madrid (UCM) and Instituto de Investigación Hospital 12 de Octubre (i+12), Madrid, Spain; ^2^ Neurovascular Pathophysiology Group, Centro Nacional de Investigaciones Cardiovasculares Carlos III (CNIC), Madrid, Spain

**Keywords:** stroke, fibrin, NETs, neuroprotection, TLR4

## Abstract

Stroke is one of the most prevalent diseases worldwide caused primarily by a thrombotic vascular occlusion that leads to cell death. To date, t-PA (*tissue-type plasminogen activator*) is the only thrombolytic therapy approved which targets fibrin as the main component of ischemic stroke thrombi. However, due to its highly restrictive criteria, t-PA is only administrated to less than 10% of all stroke patients. Furthermore, the research in neuroprotective agents has been extensive with no translational results from medical research to clinical practice up to now. Since we first described the key role of NETs (*Neutrophil Extracellular Traps*) in platelet-rich thrombosis, we asked, first, whether NETs participate in fibrin-rich thrombosis and, second, if NETs modulation could prevent neurological damage after stroke. To this goal, we have used the thromboembolic *in situ* stroke model which produces fibrin-rich thrombotic occlusion, and the permanent occlusion of the middle cerebral artery by ligature. Our results demonstrate that NETs do not have a predominant role in fibrin-rich thrombosis and, therefore, DNase-I lacks lytic effects on fibrin-rich thrombosis. Importantly, we have also found that NETs exert a deleterious effect in the acute phase of stroke in a platelet-TLR4 dependent manner and, subsequently, that its pharmacological modulation has a neuroprotective effect. Therefore, our data strongly support that the pharmacological modulation of NETs in the acute phase of stroke, could be a promising strategy to repair the brain damage in ischemic disease, independently of the type of thrombosis involved.

## Introduction

Stroke affects 15 million people per year due to a thrombus producing an abrupt decrease in blood flow supply to the brain that leads to the initiation of an inflammatory cascade and that, ultimately, will drive to cell death if the blood flow is not restored. Only two treatment regimens are currently approved by the FDA and the EMA: pharmacological thrombolysis using t-PA (tissue plasminogen activator) and mechanical thrombectomy. Unfortunately, both treatments have, as limitations, a low rate of arterial recanalization and the phenomenon of hemorrhagic transformation (HT), among others (1, 2). Indeed, recanalization of the occluded artery is limited to a small proportion of patients (3): specifically, due to highly restricted criteria, t-PA can only be administrated to less than 10% of stroke patients and less than 50% of t-PA-treated patients have successful recanalization (4). In this context, the research in fibrinolytic but also neuroprotective agents in stroke has been extensive with no translational results from medical research to clinical practice up to now.

Stroke elicits an extensive inflammatory response in brain with the recruitment of circulating leukocytes (5). Among these, neutrophils are the most abundant white blood cells that are rapidly recruited to the site of injury providing an effective immune response. Neutrophils are known to release their intracellular content in a web-like structure called Neutrophil Extracellular Traps (NETs). NETosis is a complex cellular process characterized by the release of DNA decorated with granular proteins (MPO or elastase) and histones in response to microbial infection (6). The release of these traps is dependent on the citrullination of histones by the peptidyl arginine deiminase type IV (PAD) enzyme and decondensation of chromatin (7). In addition to their bactericidal effect, NETs have also been documented in sterile inflammation, atherosclerosis, or ischemia/reperfusion injury (8, 9). Additionally, NET formation has been related to platelet TLR4 (Toll-like receptor 4) activation, but its role in the setting of thrombotic stroke is unknown (10). 

Importantly, it has been widely described that stroke thrombi are mainly composed by fibrin, platelets, red blood cells, and NETs (11, 12) Since Fuchs and cols. described for the first time the relationship between NETs and thrombosis in a platelet-dependent manner (13), many researchers including us have been trying to understand how platelets and NETs interact to lead to thrombus formation (14–16), (16). Indeed, our group recently described that inhibition of NETs through different pharmacological approaches leads to an effective lysis of platelet-rich thrombi in a platelet TLR4-dependent manner (17). However, it remains unknown whether NETs play a crucial role in fibrin-rich thrombi, as well as in tissue damage in the acute phase of stroke.

Therefore, in this study, we decided to elucidate 1) the role of NETs in fibrin-rich thrombosis, and 2) whether NET formation affects outcome in acute stroke.

## Materials And Methods

### Animals

All experiments were performed in C57bl/6 male mice, 8-12 weeks-old and weighting 20-25g (Harlan, Spain). Transgenic mice that express the Cre recombinase enzyme under platelet factor 4 promoter (PF4-Cre) were kindly donated by Dr. Andrés Hidalgo. Transgenic mouse was crossed with TLR4^loxP/loxP^ mice, kindly donated by from Prof. Timothy Billiar (University of Pittsburgh, USA), to delete TLR4 in platelets.

Mice were kept in ventilated cages at 22°C in a 12h light/dark cycle and 35% humidity with *ad libitum* access to food and water. All procedures were performed in accordance with the European Communities Council Directive (86/609/EEC) and approved by the Ethics Committee on Animal Welfare of University Complutense (PROEX number 016/18) and are reported according to ARRIVE guidelines. A special effort was made to reduce the number of animals used in the study and to provide them with the most comfortable conditions possible.

### Treatments

In the first set of experiments saline, t-PA (10mg/kg intravenously), or DNase-I (50µg in 250µl of saline intraperitoneally and a second dose of 10µg intravenously) were administrated 3 hours after the thromboembolic occlusion (17).

For permanent middle cerebral artery occlusion (pMCAO) set of experiments, either vehicle or DNase-I treatments were administered 10 minutes after MCA occlusion. Peptidylarginine deiminase (PAD) inhibitor N-α-benzoyl-N5-(2-chloro-1-iminoethyl)-l-ornithine amide (Cl-amidine, Cayman) was used to inhibit NET formation (18). Cl-amidine was dissolved in PBS and a dosage of 10mg/kg was intravenously administered 20 min before and after MCAO and 24 hours after the occlusion. A second group of mice received similar volume of PBS alone at the same time points as control group.

### Surgical Procedures

All experiments were performed and quantified in a randomized fashion by investigators blinded to specific conditions for prevention of bias. Two different animal models of ischemic stroke were used: occlusion of the middle cerebral artery occlusion by thromboembolic *in situ* model and permanent middle cerebral artery occlusion by ligature (pMCAO). In both models, mice were anesthetized and maintained during surgery at 1-2% isoflurane in a mix of O_2_ and synthetic air (O_2_/N_2_; 0.2/0.8 L/min). Body temperature was maintained at physiological levels with a heating blanket during surgery and anaesthesia recovery. Following surgery, subjects were returned to their cages and allowed free access to water and food. Animals were sacrificed by an overdose of isoflurane 24h after the ischemic insult.

#### Thromboembolic *In Situ* Middle Cerebral Artery Occlusion (MCAO)

To recapitulate fibrin-rich thrombotic stroke, a thromboembolic *in situ* MCAO model was used. Briefly, the skin between the left ear and eye was cut and then temporal muscle retracted to perform a small craniectomy over the artery bifurcation. Then, the dura was removed and thrombin (Molecular Innovations, 1 µl, 2 UI) was injected into the lumen of the MCA to produce a fibrin-rich clot (19). A laser doppler was used in order to measure the cerebral perfusion (PeriFlux System 5000; Perimed AB, Sweden) placed over the parietal branch of the MCA. The occlusion was considered to be appropriate with a drastic fall of brain perfusion (reduction of 50%). A recovery of brain perfusion beyond 50% of the basal level in the ischemic territory was considered as a successful reperfusion of the occluded vessel.

#### Ligature Model by Permanent Middle Cerebral Artery Occlusion (pMCAO)

Left common carotid artery (CCA) and left middle cerebral artery (MCA) were exposed and occluded permanently by ligation as previously described (pMCAO) (20). Complete interruption of blood flow was confirmed under an operating microscope.

### Neurobehavioral Assessment

Modified neuroseverity score (mNSS) was used to measure functional deficits induced by MCAO in mice 24h after the ischemic insult. Sensory and motor deficits were evaluated through the neuroseverity score adapted for mice (21). A minimum score of 7 reflects most severe neurological deficit and a maximum score of 21 reflects absence of deficits.

### Determination of Brain Infarct Size

Infarct volume was assessed at 24h using magnetic resonance imaging (MRI). MRI was performed using a BIOSPEC BMT 47/40 (Bruker, Ettlingen, Germany). T2-weighted images were acquired, and infarct volume was determined as described (22).

### Cytokine Determination by Cytometric Bead Array (CBA)

Blood extraction was performed by cardiac puncture in the right ventricle of euthanized mice. Plasma cytokines levels were measured 24h after pMCAO with BD CBA Mouse T_h_1/T_h_2/T_h_17 Cytokine kit (BD Biosciences, San Jose, CA, USA) according to manufacturer´s instructions. The kit was used for the detection of mouse IL-2, IL-4, IL-6, IL-10, IFN-γ, TNF-α and IL-17A in a single sample simultaneously. This kit provides a mixture of 7 capture beads with distinct size and fluorescent intensities that have been conjugated with specific antibodies for each cytokine. Four-color flow cytometric analysis was performed using a FACSCalibur flow cytometer (Becton Dickinson). Data was acquired with the BD CellquestTM PRO and analyzed using the FCAP Array™ software. Protein concentration was expressed as pg/ml.

### Immunofluorescence on Mouse Brain Sections

Mice were anesthetized and perfused intracardially with phosphate buffer (pH 7.4) followed by paraformaldehyde (PFA, 4% in phosphate buffer). The brain was removed, post-fixed with PFA overnight, cryoprotected in 30% sucrose, frozen and 15 μm-thick sections were obtained in the cryostat. Sections were first incubated for 2 h with blocking solution (BSA 0.5%, normal serum 10% and Triton X-100 0.25% in PBS); next, primary antibodies were incubated overnight at 4°C: histone-3 citrulline (1:400, Abcam), elastase (1:500, Abcam) and NIMP-R14 (1:200, Abcam). Then, sections were incubated for 2 h at room temperature with secondary antibodies (Alexa Fluor-488, -532, -647, Abcam). Immunoreaction controls were always carried out by omission of the primary antibodies. Sections were observed under a confocal laser microscope with ×63 oil lens. (LSM710; Zeiss, Germany) (17).

### Quantification of NETs

NETs were identified by NIMP-R14, neutrophil elastase and histone-3 citrulline antibodies. The number of NETs present in the ipsilesional brain was counted in 10 randomly selected fields with confocal laser microscope (LSM710; Zeiss, Germany). The sum of them (a total number of 10 fields) was expressed as the number of NIMP-R14/Elastase/Cit-H3 positive cells (23).

### Platelet Aggregation Assay

Mouse blood was drawn from the inferior vena cava of anesthetized mice, using a 25-gauge needle, into clexane (100U). Platelets were isolated as previously described (24). Next, platelet aggregation was evaluated by transmission aggregometry light in a 96-well plate. Briefly, isolated platelets were adjusted to a concentration of 3 x 10^8^/mL and subsequently stimulated with different thrombin concentrations (0.01-1 U/mL). The plate was analyzed in a plate reader of Infinite F-50 plates (TECAN) with a 405 nm excitation filter for 20 minutes (10 cycles with 1 minute of agitation between each cycle) (25).

### Statistical Analysis

Data were expressed as mean ± SEM for the indicated number of experiments. Statistical analysis was performed with Prism4 (GraphPad Software, La Jolla, CA) using parametric or nonparametric unpaired student t-test or ANOVA comparisons with a p value <0.05 was considered statistically significant.

## Results

### Delayed Administration of DNase-I Improves Stroke Outcome After Thromboembolic MCAO Despite Lack of Reperfusion

**Figure 1:**
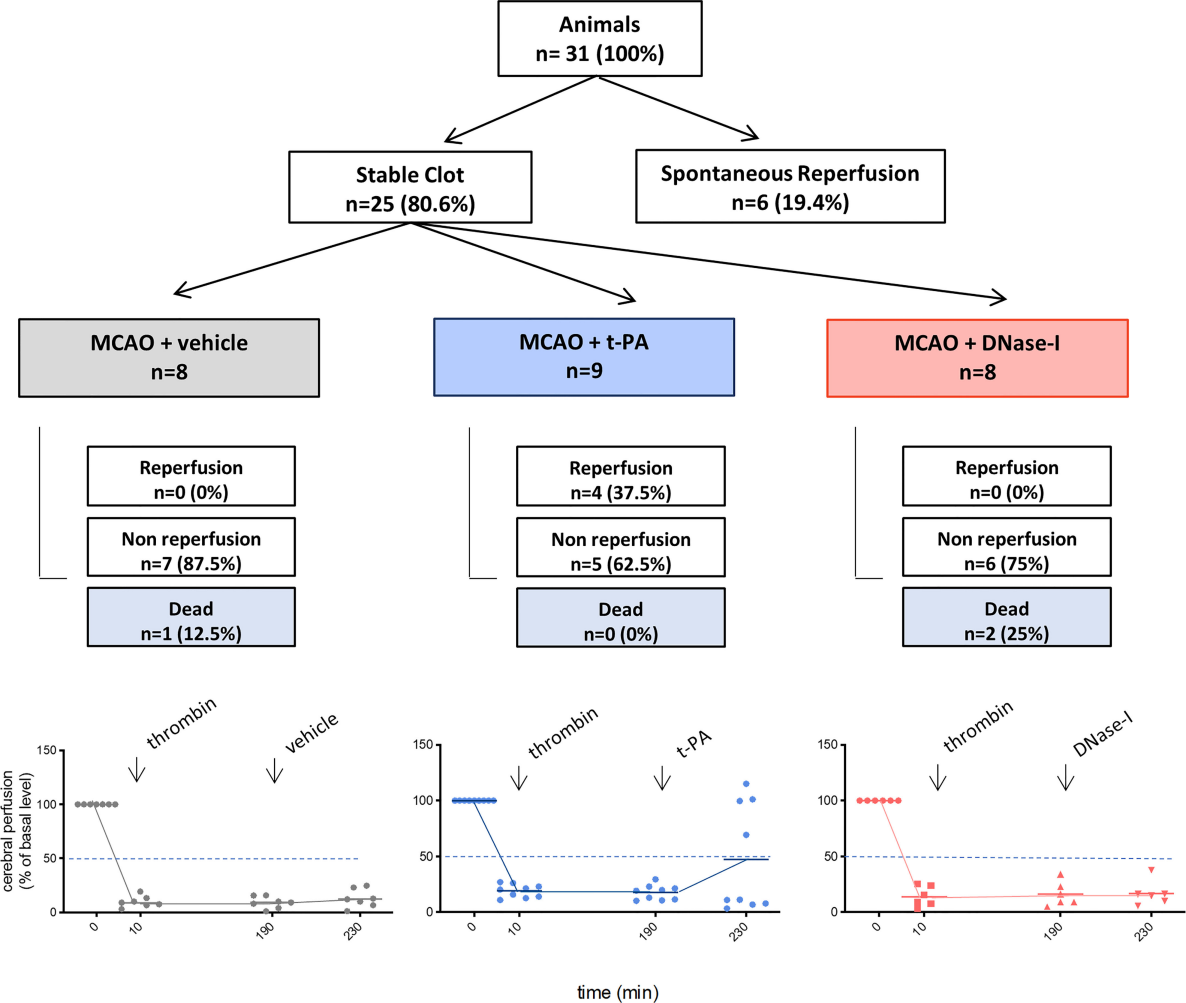
Flow chart of the study. MCAO indicates middle cerebral artery occlusion by the thromboembolic *in situ* stroke model; and tPA, tissue-type plasminogen activator.

**Figure 2:**
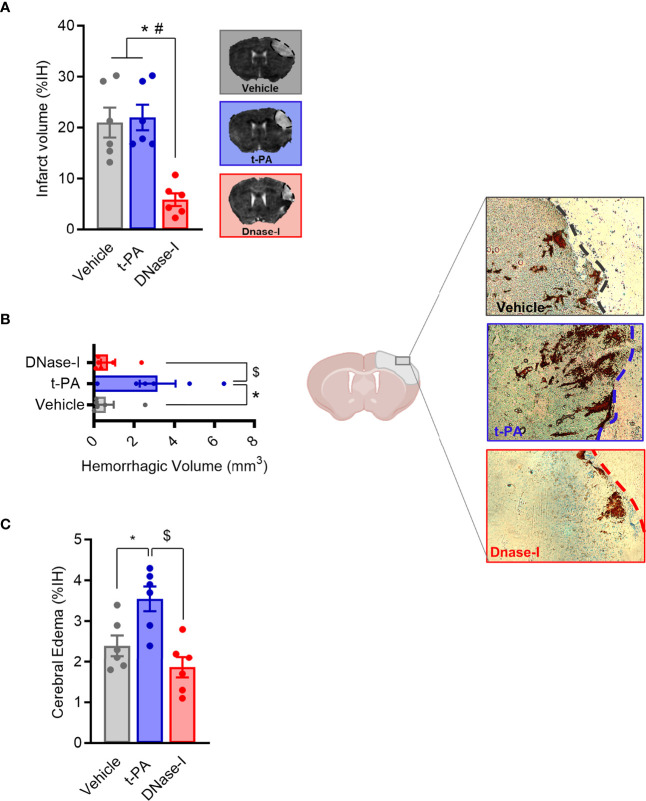
Effect of t-PA and DNase-I treatments administrated 3 hours thromboembolic occlusion. **(A)** Infarct volume determined 24 hours after the occlusion. **(B)** Hemorrhagic volume (mm^3^). **(C)** Cerebral edema. Data are mean± SEM (n = 6 *p < 0.05 vs vehicle; ^#^p < 0.05 tPA vs DNase-I; ^$^p < 0.05 tPA vs DNase-I).

One of the main components of ischemic stroke thrombi is fibrin and t-PA is the only FDA approved drug for stroke patients. In a previous study we showed how early tPA administration recanalizes the occluded vessel and improves outcome after thromboembolic MCAO (26). We now corroborate that, in contrast, the delayed administration of t-PA, despite being able to effectively recanalize high-fibrin content thrombi resulting from the *in situ* thromboembolic stroke model (**Figure 1**), does not affect infarct volume (n=6; **Figure 2A**) and causes bleeding and cerebral edema because of the HT phenomenon (n=6; p<0.05; **Figures 2B, C**). At the same time, we demonstrate that the delayed administration of DNase-I (a promoter of NETs degradation) is not able to effectively recanalize the occluded artery in this fibrin-rich thrombotic model (**Figure 1**), suggesting a scarce contribution of NETs to fibrin-rich thrombi composition.

Importantly, despite non-reperfusion, DNase-I administration significantly reduced the infarct volume when compared to either vehicle or t-PA-treated mice (n=6; p<0.05; **Figure 2A**) and, unlike t-PA, produces neither increased cerebral edema nor HT (n=6; p<0.05; **Figures 2B, C**), this demonstrating that DNase-I does not exacerbate damage to the blood-brain barrier.

### Early Administration of DNase-I Reduces Infarct Size and Improves Stroke Outcome After Ligature-Induced Permanent MCAO

**Figure 3:**
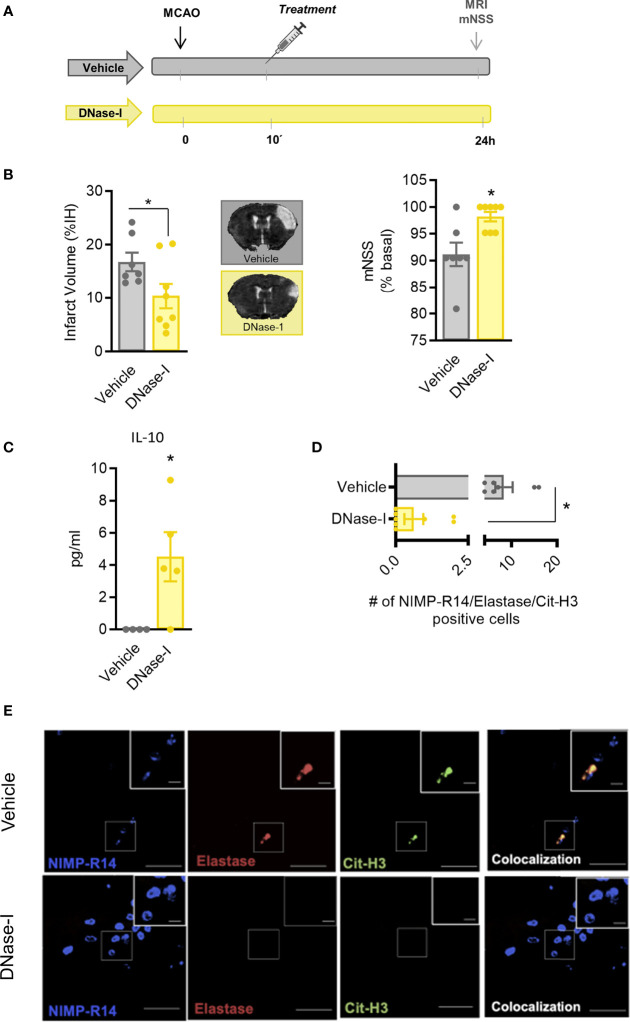
Effect of DNase-I treatment after stroke. **(A)** Design of the study. **(B)** Infarct volumes determined 24 hours after the occlusion. **(C)** Effect of DNase-I on plasma concentration of IL (interleukin)-10 24 hours after the occlusion. **(D)** Number of NIMP-R14, elastase and Cit-H3-positive cells. **(E)** Representative images. Data are mean± SEM (n = 4-8 *p < 0.05 vs vehicle).

These results support that DNAse-I exerts protective effects which are independent of blood vessel recanalization. To investigate the mechanisms involved, the MCAO by ligature model was selected to produce a permanent occlusion of the middle cerebral artery. Thus, pMCAO produced an infarct lesion, as assessed 24 h after the occlusion using magnetic resonance imaging (MRI) and caused neurological deficits (**Figure 3B**). Again, the early administration of DNase-I significantly reduced the infarct volume and was associated with a better functional outcome after pMCAO (n=7-8; p<0.05; **Figure 3B**)

In order to explore further the mechanisms involved in the protective effect of DNase-I, we performed a quantitative analysis of different plasma cytokines using a customised CBA. Animals treated with DNase-I showed a significant increase in plasma protein levels of IL-10 24 h after the ischemic insult, when compared with vehicle group (n=4-5; p<0.05; **Figure 3C**). No significant differences were found between groups in IL-2, IL-4, IL-6, IFN-γ, TNF and IL-17A protein levels (data not shown).

To identify signs of NETosis in the ischemic brain, we used different antibodies against neutrophil (NIMP-R14), elastase and citrulline histone 3 (Cit-H3). Our data show that early administration of DNase-I significantly reduces the presence of neutrophils with Cit-H3 and neutrophil elastase positive staining in the ischemic cortex 24 h after stroke (n=7-8; p<0.05; **Figures 3D, E**).

### NETs Inhibition by Cl-amidine Is Protective After Stroke by Ligature-Induced Permanent MCAO

**Figure 4:**
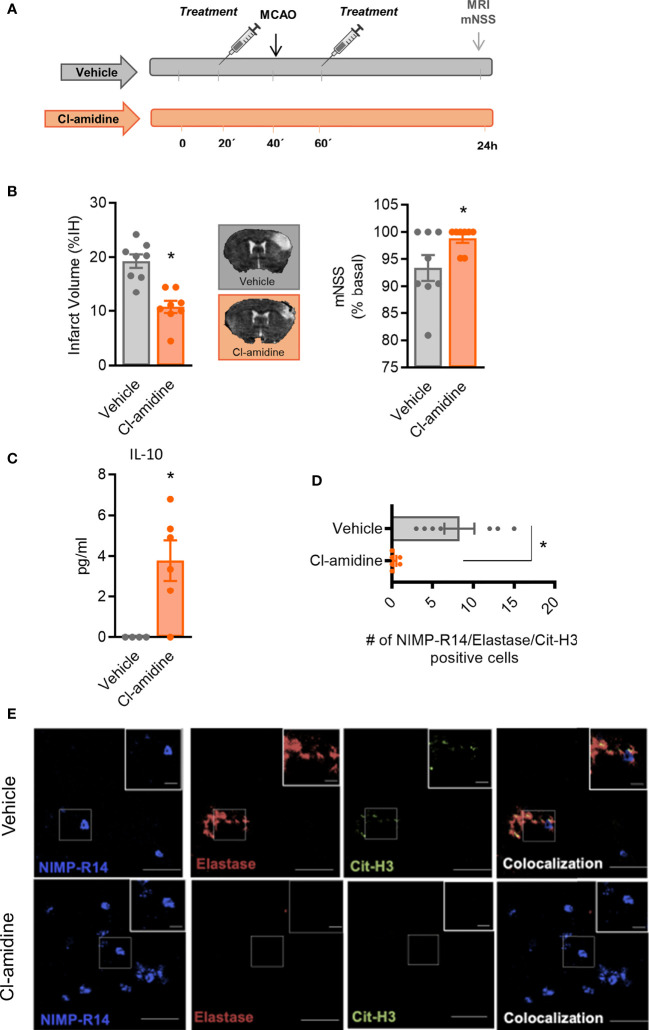
Effect of Cl-amidine treatment after stroke. **(A)** Design of the study. **(B)** Infarct volumes determined 24 hours after the occlusion. **(C)** Effect of Cl-amidine on plasma concentration of IL (interleukin)-10 24 hours after the occlusion. **(D)** Number of NIMP-R14, elastase and Cit-H3-positive cells. **(E)** Representative images. Data are mean± SEM (n = 4-8 *p < 0.05 vs vehicle).

Because DNase-I could not only depredate the DNA released by neutrophil extracellular traps but also the DNA released by dying cells after stroke, and to further confirm the detrimental role of NETs after experimental stroke, we treated animals with amidine to inhibit NETs formation (18) (**Figure 4A**). Compared to vehicle-treated animals, mice treated with Cl-amidine showed reduced infarct volume as well as a significant improvement in functional outcome scores (n=8; p<0.05; **Figure 4B**).

Our results also indicate an anti-inflammatory profile in Cl-amidine-treated mice as shown by an increase in systemic cytokine levels of IL-10 after stroke when compared to vehicle treated ones (n=4-6; p<0.05; **Figure 4C**).

To further confirm the effect of Cl-amidine in NET content in the brain after ischemia, NETs immunostaining was examined using different antibodies. As expected, the administration of Cl-amidine significantly decreased NET content in ischemic brain when compared to vehicle-treated mice (n=6-7; p<0.05; **Figures 4D, E**).

### Genetic Deletion of Platelet TLR4 Induces Neuroprotection After Stroke by Ligature-Induced pMCAO

To further explore the *in vivo* mechanism of NETs formation and, considering that platelet TLR4 plays an instrumental role in platelet-rich thrombosis mediated by NETosis, we hypothesized that TLR4 on thrombocytes might have a role in NETs formation in experimental stroke by pMCAO.

**Figure 5:**
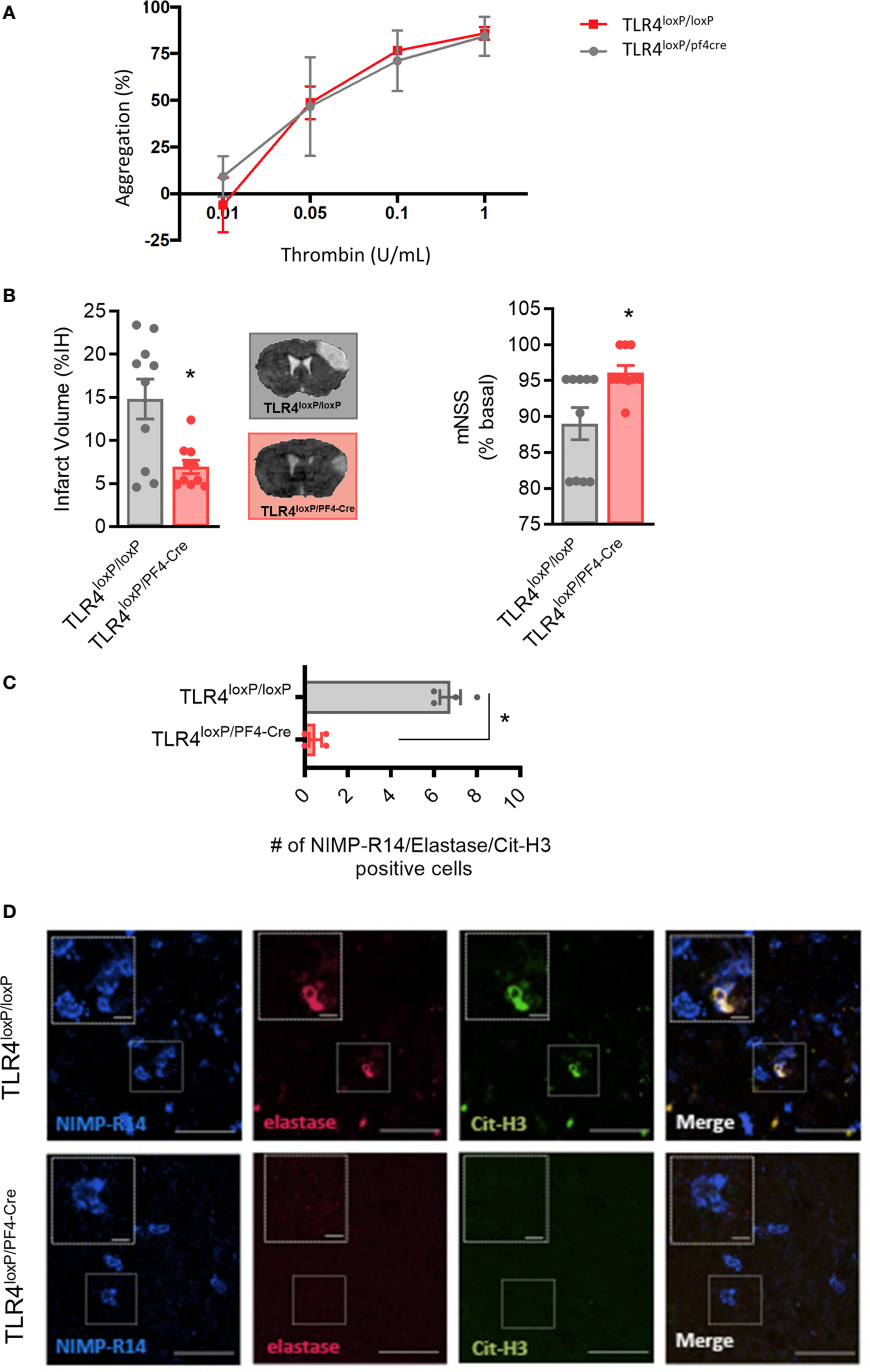
Effect of deletion of TLR4 on platelets after stroke. **(A)** Effect of TLR4 deletion on platelet aggregation **(B)** Infarct volumes determined 24 hours after the occlusion. **(C)** Number of NIMP-R14, elastase and Cit-H3-positive cells. **(D)** Representative images. Data are mean ± SEM (n = 4-10 *p < 0.05 vs TLR4^loxP/loxP^).

To test our hypothesis, we used transgenic mice that express the Cre recombinase enzyme under the PF4 promoter (PF4), crossed with TLR4^loxP/loxP^ mice, to obtain a deletion of TLR4 selectively in platelets (27). First, ablation of TLR4 on platelets did not affect platelet function when compared with TLR4^loxP/loxP^ mice (n=6; **Figure 5A**). More importantly, our data show that TLR4^loxP/pf4-cre^ mice have smaller lesion volumes compared with their respective controls after permanent MCAO by ligature, accompanied by a better functional outcome (TLR4^loxP/loxP^; n=10; p<0.05; **Figure 5C**).

To further confirm the role of platelet TLR4 in NET content in the brain after ischemia, NETs immunostaining was performed. Of note, the genetic deletion of TLR4 on platelets significantly decreased NET content in ischemic brain when compared to control mice (n=4; p<0.05; **Figures 5C, D**). Overall, these data support that platelet TLR4 has a crucial role in stroke damage in a NET-dependent manner.

## Discussion

Stroke is caused primarily by a thrombotic vascular occlusion that initiates a cascade of events that ultimately lead to neuronal cell death. Recent data suggest that the efficacy of either thrombectomy or intravenous thrombolysis is affected by thrombi composition; more specifically it has been found that fibrin-rich clots were associated with less favourable clinical outcomes (28). Since NETs have a predominant role in thrombosis (12) and since we have previously demonstrated that these extracellular traps play a key role in platelet-rich thrombosis in cerebral ischemia (17), we next want to explore their role in high-fibrin rich thrombosis. Our results demonstrate for the first time that NETs are not a main component in stroke thrombi with high fibrin content (**Figure 1**). This is in accordance with previous results showing that fibrin content in thrombus is associated with a decrease in neutrophil accumulation and NET formation (29).

At the same time, on the one hand, we have demonstrated that DNase-I was not able to effectively reperfuse the MCA, at least within the first 3 hours after the thromboembolic occlusion, whereas tPA did (**Figure 1**). However, we cannot discard the possible recanalization within the next following 20 hours previous to the sacrifice, issue that should be explore in future studies. This is in agreement with previous results showing that lower levels of NETs in thrombi produced by fibrin makes the administration of DNase ineffective (24). On the other hand, despite non-reperfusion, a novel and important observation of our study is that the late administration of DNase-I had a protective effect shown as an improvement in infarct volume and in stroke outcome (**Figure 2**). At the mechanistic level, this could be due to a direct protective effect of DNase-I and/or to a decrease in NET-dependent microthrombotic “no-reflow” phenomena that could contribute to ischemic injury.

In this context, DNAse-I, by favouring the degradation of NETs, could have direct protective properties since NETs have been implicated in brain damage: specifically, neutrophils, as the first group of cells infiltrating the damaged brain tissue, produce NETosis in brain parenchyma and peripheral blood that aggravate inflammation and subsequent brain damage after stroke (30, 31). NETs are also associated with severity and mortality in patients (32). In fact, we have demonstrated that NET degradation has a protective effect as measured by the peripheral levels of IL-10 (**Figure 3**) which is in accordance with what others have observe not only in stroke but in sepsis models (33, 34). However, the fact that we didn’t observe any significant difference among treatments in other cytokines should be deeply explore in future studies.

Furthermore, hyperglycemia, which increases NETs formation, also exacerbates ischaemic brain damage (35). Finally, we and others have shown that drugs that prevent NET formation protect against ischemic damage in several stroke models (**Figure 4**) (16, 36). However, not only neutrophils released their content to extracellular space but also macrophages (METs), eosinophils (ETs) or even mast cells released their intracellular content in a ETosis cell-death way (37–39). In this context, even though we have target NETs with neutrophil specific antibodies, we cannot exclude the detrimental role of other form of ETosis in the acute phase of stroke, a fact that should be explore in future studies.

Secondly, DNAse-I, by preventing the formation of microthrombosis and “no-reflow” phenomena, may have also neuroprotective effects. It is well known that clot removal and vessel recanalization do not always go along with tissue reperfusion, a phenomenon called “no-reflow” that occurs at both the cardiac and cerebral levels (40). Only about 50% of t-PA treated patients have successful reperfusion. It is likely that multiple mechanisms might contribute to the microvascular no-reflow phenomenon, including endothelial cell dysfunction, microthrombosis, neutrophil accumulation and NETs formation, among others (41–43). In this context, several studies have shown the existence of microthrombi-induced occlusions in small-calibre vessels adjacent to the infarcted area (41, 44). Microvascular obstruction of 20-30% of capillaries in the core and penumbra of the infarct has been shown to be produced by neutrophils adhering to distal capillary segments; furthermore, removal of circulating neutrophils using an anti-Ly6G antibody restores microvascular perfusion without increasing the rate of hemorrhagic complications (41). Of note, the involvement of NETs in microthrombosis phenomena has been demonstrated (43). Therefore, NETs could be participating in the formation of small thrombi generated after a large vessel occlusion, in such a way that both DNase-I (by favouring the degradation of NETs) and Cl-amidine (by inhibiting the formation of NETs) could be beneficial in resolving the microthrombosis phenomena associated with stroke, a fact that deserves to be studied in more detail.

Interestingly, we have also demonstrated the deleterious role of platelet TLR4 in brain ischemia caused by non-platelet-rich thrombosis (**Figure 5**). We had previously demonstrated that TLR4 in platelets is determinant for the photothrombotic formation of a stable platelet-rich thrombus which, in turn, depended on NETs (16); now, we demonstrate that platelet TLR4 is also determinant in brain damage produced by occlusions/thrombi in which NETs do not play a relevant role. These data support the role of NETs-induced microthrombosis in ischemic damage, since platelet TLR4 ultimate triggers the NETosis process (10, 17).

DNAse-I treatment did not affect the blood-brain barrier since, as opposed to t-PA, its delayed administration produced neither cerebral edema nor HT (**Figure 1**). This finding supports that DNase-I could be a safe therapy, deprived of the severe side effects associated with tPA-induced fibrinolysis.

Our study has some limitations. One is that the experiments have been performed in young animals, without the co-morbidities present in many stroke patients. In addition, we included only male mice to compare our results with the previous work done in this stroke model. Therefore, co-morbidities, sex-related differences and mechanisms involved in neuroprotection and no-reflow after stroke deserve further investigations.

Overall, our results support the important role of NETs in cerebrovascular disease and point to the involvement of the platelet TLR4 receptor in this process, thus providing new avenues for the treatment of the acute phase of stroke through the inhibition and/or degradation of the extracellular traps released by the neutrophil.

## Data Availability Statement

The original contributions presented in the study are included in the article/supplementary material. Further inquiries can be directed to the corresponding authors.

## Ethics Statement

The animal study was reviewed and approved by Ethics Committee on Animal Welfare of University Complutense (PROEX number 016/18).

## Author Contributions

CP-M, VD-L, AG-C, MM, and IL designed the research studies. CP-M, VD-L, AG-C, and MIC conducted the experiments and/or acquired the data. CP-M, VD-L, AG-C, MIC, MM, and IL contributed to the analysis and/or interpretation of the results. CP-M, MM, and IL wrote the manuscript. All authors contributed to the article and approved the submitted version.

## Funding

This work was supported by grants from Instituto de Salud Carlos III and co-financed by the European Development Regional Fund “A Way to Achieve Europe” PI20/00535 and RETICS RD16/0019/0009 (IL), from Regional Madrid Government B2017/BMD- 3688 (IL), from Spanish Ministry of Science and Innovation PID2019-106581RB-I00 (MÁM), from Leducq Foundation for Cardiovascular Research TNE-19CVD01 (MÁM), from Fundación La Caixa HR17_00527 (MM). The CNIC is supported by the Instituto de Salud Carlos III (ISCIII), the Ministerio de Ciencia e Innovación (MCIN) and the Pro CNIC Foundation, and is a Severo Ochoa Center of Excellence (SEV-2015-0505).

## Conflict of Interest

The authors declare that the research was conducted in the absence of any commercial or financial relationships that could be construed as a potential conflict of interest.

## Publisher’s Note

All claims expressed in this article are solely those of the authors and do not necessarily represent those of their affiliated organizations, or those of the publisher, the editors and the reviewers. Any product that may be evaluated in this article, or claim that may be made by its manufacturer, is not guaranteed or endorsed by the publisher.
